# PD06 Automated Systems For Hospital Inpatient Safety: A Cost-Utility Analysis

**DOI:** 10.1017/S0266462324002757

**Published:** 2025-01-07

**Authors:** Emily Holmes, Huw Lloyd Williams, Dyfrig Hughes, Elke Naujokat, Bernd Duller, Christian Subbe

## Abstract

**Introduction:**

Deployment of an electronic automated advisory vital signs monitoring and notification system to signal clinical deterioration is associated with significant improvement in clinical outcomes. This study aimed to estimate the incremental cost per quality-adjusted life-year (QALY) gained with an electronic automated advisory notification system, compared with standard care.

**Methods:**

A decision analytic model was developed to estimate the cost effectiveness of an electronic automated advisory notification system, compared with standard care, in adults admitted to a district general hospital. Analyses considered the following: (i) cost effectiveness (cost/event avoided) based on a before-and-after study (n=3,787) that recorded rates of acute myocardial infarction, pulmonary embolism, acute pulmonary edema, respiratory failure, stroke, severe sepsis, acute renal failure, cardiopulmonary arrest, admission to the intensive care unit, and death; and (ii) the cost utility (cost per QALY) over a lifetime horizon extrapolated using published data. The analysis was conducted from the perspective of the National Health Services (NHS) in the UK.

**Results:**

The automated notification system was more effective (2.7 fewer events per 100 patients) and provided cost savings of −GBP12.17 [−EUR14.07] per patient admission (95% CI: −GBP182.07 [−EUR211.20], GBP154.80 [EUR179.57]). The automated notification system was dominant over a lifetime horizon, demonstrating a positive incremental QALY gain (0.0287 QALYs, equivalent to approximately 10 days of perfect health) and a cost saving of −GBP55.35 (−EUR64.02). At a threshold of GBP20,000 per QALY (EUR23,126), the probability of automated monitoring being cost effective in the NHS was 0.81. The increased use of cableless sensors may reduce cost-savings, but the intervention remained cost effective at 100 percent usage (incremental cost-effectiveness ratio GBP3,107 per QALY [EUR3,594 per QALY]).

**Conclusions:**

An automated notification system for adult patients admitted to general wards appears to be a cost-effective strategy in the NHS. The analysis suggests that adopting this technology could be good use of scarce resources. The impact of automated monitoring solutions on staffing warrants further exploration and may show additional value in adopting such technology.

